# P-1974. Surface Protein Profiling of Extracellular Vesicles: A Novel Biomarker Approach for Long COVID Diagnosis?

**DOI:** 10.1093/ofid/ofae631.2132

**Published:** 2025-01-29

**Authors:** Gentaro Ikeda, Prasanna Jagannathan, Mario Leki, Hiroyuki Inoue, Vanessa El Kamari, Arya V Dadhania, Mitchell Miglis, Robert W Shafer, Linda N Geng, Phillip C Yang, Hector F Bonilla

**Affiliations:** Stanford School of Medicine, Stanford, California; Stanford University, Stanford, California; Stanford School of Medicine, Stanford, California; Stanford School of Medicine, Stanford, California; Stanford School of Medicine, Stanford, California; Stanford School of Medicine, Stanford, California; Stanford School of Medicine, Stanford, California; Stanford School of Medicine, Stanford, California; Stanford School of Medicine, Stanford, California; Stanford School of Medicine, Stanford, California; Stanford University School of Medicine, Palo Alto, California

## Abstract

**Background:**

Extracellular vehicles (EVs) are lipid bilayer vesicles secreted by all cell types into the circulation. EVs carry an array of biomolecules indicative of their origin cells and act as cargo vesicles capable of delivering proteins, mitochondria, and viral particles. However, whether long COVID affects the profile of plasma EVs remains unknown.

**Methods:**

Plasma samples were selected from the Stanford repositories of Long COVID participants—seventy-six plasma samples from patients with documented SARS-CoV-2 infection. Twenty-five participants had full recovery (10 months post-infection, Stanford Lambda Trial), 24 patients with Long COVID with CFS phenotype and 27 with ME/CFS from the Stanford Long COVID clinic. Plasma EVs were isolated using size-exclusion columns (qEV, Izon Science). The positivity for surface markers and mitochondrial membrane potentials of the plasma EVs were evaluated by flow cytometry. Samples were stained with pre-titrated volumes of the following fluorochrome-conjugated monoclonal antibodies: CD45 leukocytes, CD3/T cells, CD20/B cells, CD14/Monocytes, CD16/NK cells, CD144/endothelial cells, CD41a/Platelets, CD56/neural cell adhesion molecule (NCAM), and Mitotracker deep red/mitochondrial membrane potential.

**Results:**

The total EV counts and the proportion of mitochondria-positive were decreased in Long COVID group compared to the COVID recovery group (5.02 x 10^6^ vs. 1.92 x 10^7^ /mL, and 42.7 vs. 56.7%, respectively). Compared with the recovery group, Monocyte and NK cell-derived EVs were significantly increased, whereas Endothelial cell-derived EVs were decreased in the Long COVID group. The EVs from the Long COVID group consistently exhibited lower positivity of mitochondrial membrane potential regardless of the parental cells. No difference was observed in plasma EVs derived from NCAM positive cells. NK cell proportion is the only difference between LC/CSF and CSF (P= 0.046).

**Conclusion:**

Patients with Long COVID showed alterations in plasma EV profiles. These findings may offer insights into the pathogenesis of long COVID.

**Disclosures:**

All Authors: No reported disclosures

